# Boosting Digital Health Engagement Among Older Adults in Hong Kong: Pilot Pre-Post Study of the Generations Connect Project

**DOI:** 10.2196/69611

**Published:** 2025-05-08

**Authors:** Aaron Wan Jia He, Runqi Yuan, Tzu Tsun Luk, Kelvin Man Ping Wang, Sophia Siu Chee Chan

**Affiliations:** 1 School of Public Health, Li Ka Shing Faculty of Medicine, University of Hong Kong, Rm 520, 5/F, Academic Building, No. 3 Sassoon Road, Pokfulam, Hong Kong, China (Hong Kong), 852 39176610; 2 School of Nursing, Li Ka Shing Faculty of Medicine, University of Hong Kong, Hong Kong, China (Hong Kong)

**Keywords:** older adults, eHealth literacy, home-based intervention, intergenerational learning, health promotion

## Abstract

**Background:**

Older adults’ utilization of digital health care remains low despite a high demand for regular health services. Easily accessible eHealth interventions designed for older adults are needed.

**Objective:**

This study aimed to examine the feasibility and effectiveness of an intergenerational, home-based eHealth literacy intervention package on older adults in Hong Kong.

**Methods:**

In this study, 101 older adults (n=64, 63.4% female) with a median age of 80 (IQR 77-85) years received an intergenerational, home-based eHealth literacy intervention package, delivered by trained university student interventionists. The intervention (median 60, IQR 40.8-70 minutes) included personalized guidance on using mobile health apps, QR code scanners and instant messaging, and access to online health information, along with recommendations for physical and mental well-being. Following the intervention, a daily health-coaching message was sent to older adults via WhatsApp for 14 days. eHealth literacy, health, and lifestyle were assessed at baseline and at a 2-week follow-up using paired *t* tests.

**Results:**

Retention rate for the 2-week follow-up was 70.3% (71/101). Compared to baseline, eHealth literacy scores increased by 2.39 points (*P*=.11; Cohen *d*=0.20), and daily smartphone use rose by 0.45 hours (*P*=.07; Cohen *d*=0.05). Participants self-reported increased physical activity (50/71, 70%), more frequent viewing of health videos (43/70, 61%), and improved handwashing practices (39/71, 55%). The intervention achieved a high satisfaction rating of 4.32 out of 5.

**Conclusions:**

The intergenerational, home-based eHealth literacy intervention package was feasible and acceptable, showing promise for increasing older adults’ engagement with digital health care resources and promoting healthy behaviors. Future studies should explore longer-term effects and ways to further improve the intervention.

## Introduction

Hong Kong is undergoing a rapid demographic shift; the proportion of older adults has increased from 13.3% in 2011 to 19.6% in 2021 [1], and it is expected to reach 36.0% by 2046 [2]. Unfortunately, the rise in life expectancy comes with an increase in unhealthy years of life even among industrialized states [3], creating significant challenges for health care systems. Globally, health care expenditure has doubled in the past 2 decades, reaching 9.8% of global gross domestic product (GDP) in 2019 [4]. In 2022, Hong Kong spent 7.3% of its GDP on health care, one of the highest expenditures on health care in Asia [5]. Traditionally, people have preferred seeking care at large public hospitals even for simple health problems due to limited publicly funded primary care facilities [6]. This model of health care is unsustainable as tertiary care providers become overloaded while primary health care remains underutilized [7].

Chronic conditions and noncommunicable diseases (NCDs) affect over 70% of Hong Kong’s older adults [8], who require frequent medical care [9]. Additionally, mental health concerns such as depression are prevalent [10-12], exacerbated by social isolation [13] and loneliness [9,14,15], particularly during the COVID-19 pandemic. In response to these chronic health and mental health challenges, and in the context of primary health care reform in Hong Kong, improving older adults’ ability to access and engage with digital health care services has become essential.

eHealth literacy, defined as the “ability to seek, find, understand, and appraise health information from electronic sources” [16], has emerged as a key factor in improving health outcomes. It is thus imperative to identify and support population groups at risk of low eHealth literacy [17]. Older adults, despite their high demand for health care, often struggle to adopt digital health care tools due to a lack of confidence, interest, or familiarity with technology [18-20]. These challenges could widen the digital divide, leaving many older adults disconnected from essential eHealth resources [21]. Tailored interventions are needed to promote eHealth literacy and engage older adults more effectively with digital health care systems.

Existing studies have shown that targeted interventions can enhance eHealth literacy, promote healthy behaviors, and sustain mental health. However, these interventions often rely on convenient samples recruited from a limited pool of participants and are implemented in centralized locations, such as classrooms in community senior centers [22-25]. Such design limits accessibility for underprivileged or home-bound older adults, who may be excluded from traditional classroom-based interventions. Additionally, quantitative evidence remains scarce for home-based interventions designed to address the unique challenges faced by these populations [26-29].

This pilot study explored the effect of an intergenerational, home-based intervention package focused on improving older adults’ engagement with mobile eHealth resources, while also aiming to better their lifestyles and overall well-being. The intervention is grounded in the Information-Motivation-Behavioral Skills model, a behavior-oriented theory widely used in health promotion programs [30,31]. The Information-Motivation-Behavioral Skills model consists of 4 domains: information, motivation, behavioral skills, and behavior, positing that information, motivation, and behavioral skills directly and indirectly influence health behavior [31]. This model was chosen as it provides a comprehensive framework to address the complex factors influencing eHealth literacy in older adults. The intergenerational and home-based components of the intervention are designed to enhance its effectiveness. Intergenerational approaches have been shown to improve digital literacy and reduce social isolation among older adults by leveraging the technological proficiency of younger generations [32]. Home-based interventions are particularly beneficial for older adults as they provide a familiar and comfortable environment, reducing barriers to participation and increasing engagement [33]. By incorporating an intergenerational approach, the intervention leverages the tech-savviness of the younger generation, often referred as “digital natives,” to enhance older adults’ self-efficacy in technology use, a critical component of eHealth interventions [20]. Trained student interventionists visited older adults in their homes, providing practical knowledge on utilizing eHealth resources and adopting health-promoting behaviors. The home visits also aimed to bridge the generational gap and reduce loneliness among older adults [33]. This study aims to examine the feasibility and effectiveness of a nursing student–led, home-based eHealth intervention to better serve older adults across Hong Kong.

## Methods

This pilot study is part of the Generations Connect Project [34], a large-scale, community-based initiative aiming to train over 1000 nursing students providing community service and behavioral coaching to 10,000 older adults in Hong Kong.

### Study Design

This is a pilot, single group, pre-post study to evaluate the feasibility and effectiveness of intergenerational, home-based eHealth interventions on eHealth literacy among older adults in Hong Kong. Data collection occurred both in-person (baseline) and via telephone (2-week follow-up) in November 2022. Consolidated Standards of Reporting Trials (CONSORT) reporting guidelines and the extension to randomized pilot and feasibility trials were followed [35] (Checklist 1).

### Participants

Older adults were recruited through collaborations with local nongovernment organizations covering 18 districts across Hong Kong. Older adults were eligible if they were (1) 65 years or older, (2) spoke Chinese, and (3) were physically, mentally, and cognitively capable of engaging in simple physical tasks and (4) understanding survey questions. Older adult participants were told the availability of home-based eHealth and health behavior coaching sessions, which remove physical barriers commonly seen in centralized interventions.

Recruitment involved partnerships with community centers, senior clubs, and health care facilities. Information sessions introduced the study, followed by screening interviews for interested individuals. Those meeting the inclusion criteria were scheduled for baseline assessments. Home visits were arranged for those unable to attend group sessions.

### Ethical Considerations

This study was conducted in accordance with ethical guidelines for research involving human participants. The research protocol was approved by the Institutional Review Board of the University of Hong Kong (UW 22‐693). Informed consent was ensured through comprehensive information sheets and verbal explanations, with consent forms signed before any procedures. The consent form included information about the study’s purpose, procedures, risks, and benefits, as well as the participant’s right to withdraw at any time without penalty.

As a token of appreciation for their time and participation, older adults enrolled in the study received HKD $50 (US $6.44; HKD $1=US $0.13) gift bags as an incentive during the initial student visits.

All data collected during the study were deidentified to protect participants’ privacy. Data were initially recorded on paper and subsequently digitized using Qualtrics, with all identifying information removed. Access to the data was restricted to the research team, and all digital data were stored securely.

### Training Student Interventionists

Student interventionists were current full-time students enrolled in the Bachelor of Nursing or Master of Science in Nursing programs at the University of Hong Kong, who were fluent in Cantonese. Students were recruited through internal university emails and on-campus promotional materials such as posters. Recruited students were trained by research and clinical faculty members from the School of Nursing before attending home visits. A special-designed curriculum was developed with learning materials covering (1) the aging population and primary health care’s new journey in Hong Kong, (2) effective communication techniques with older adults, (3) assistance with digital devices and mobile-based eHealth services, (4) knowledge on NCD prevention by promoting healthy lifestyles, (5) COVID-19 preventive measures, (6) strategies for building positive mental health and well-being, and (7) home visit protocol and general safety practice guidelines.

### Measurements

Participants’ data were collected by a self-administered survey at baseline and a telephone survey at 2 weeks after the intervention. The baseline questionnaire covered demographics (9 items), physical health (4 items), exercise habits (8 items), dietary practices (5 items), smoking (3 items), alcohol consumption (1 item), psychosocial health (16 items), eHealth literacy and mobile phone and app usage (17 items), and COVID-19 preventive measures (13 items). The primary outcome was eHealth literacy assessed by the 8-item eHealth Literacy Scale (eHEALS) [30], ranging from 8 to 40, with higher scores indicating better eHealth literacy. This scale is widely regarded as reliable and is frequently used in studies involving Chinese older adults, including those in Hong Kong [36-38].

Secondary outcomes included mental well-being, evaluated by the World Health Organization–Five Well-Being Index (WHO-5 [39]; multiplied by 4 to create a convenient scale of 0 to 100; higher scores indicate better well-being), and the 3-item University of California, Los Angeles (UCLA) Loneliness Scale [40] (3 to 9; lower scores indicate lower loneliness). Physical well-being was measured by a single item (1 to 5; higher scores indicate better physical well-being). Physical activities were assessed using a modified version of the International Physical Activity Questionnaire–Short Form [41].

Feasibility outcomes included (1) the recruitment rate (number of participants enrolled/number of eligible participants) and (2) the retention rate (number of participants who completed the follow-up/number of participants enrolled). Two additional feasibility outcomes were assessed: (1) the time required to conduct assessments and deliver the intervention package and (2) student interventionists’ compliance with the intervention. Acceptability was assessed using a 5-point Likert scale measuring satisfaction with the home-based interventions and the 14-day digital follow-up message, ranging from 1 (strongly dissatisfied) to 5 (strongly satisfied).

The 2-week follow-up survey included similar items (demographics excluded) with 4 additional questions on perceived behavioral changes that occurred compared to previsit and 2 more questions on their satisfaction with the home-based interventions and 14-day digital follow-up messages.

### Home-Based Assessment, Intervention Package, and 2-Week Follow-Up

Trained student interventionists conducted assessments and delivered interventions during home visits (median 60, IQR 40.8-70 minutes). Written consent was obtained from participants before the assessment began.

The assessment process was flexible, adapting to older adults’ responses. Student interventionists conducted the baseline assessment in a semistructured manner, engaging participants in natural conversations while covering questionnaire items. Questions were read aloud to older adults, and responses were given verbally.

The intervention was similarly semistructured, covering four major themes:

1. eHealth Literacy: instructions on using WhatsApp to contact health professionals and connect with family or friends, using QR code scanners to acquire health information from printed materials, and downloading and using mobile health service apps, such as HA Go and Electronic Health Record Sharing System (eHealth).
2. NCD prevention: advice on healthy lifestyle changes, including diet, smoking cessation, alcohol moderation, and physical activities.
3. Psychosocial health: promotion of positive thinking, social engagement (adhering to COVID-19 regulations), and relaxation techniques.
4. COVID-19 prevention: education on hygiene, handwashing techniques, vaccination benefits, mask-wearing, and rapid antigen tests.

Health education leaflets with QR codes linking to online resources were provided and stored in a small blue folder. Student interventionists explained the leaflet content and tailored discussions to each older adult’s health needs. They demonstrated smartphone use, including scanning QR codes and installing health apps, ensuring participants understood and could perform these tasks independently.

At the end of the intervention, student interventionists created dedicated one-on-one WhatsApp group chats with the older adults, sending daily health-related messages for the next 14 days. Participants were encouraged to engage with the content and share their opinions, using simple responses like emojis or voice messages.

A follow-up survey was conducted via telephone by the same student interventionist 2 weeks after the visit.

### Sample Size

Given the exploratory nature of this pilot study, a sample size of 100 older adults was deemed appropriate to provide preliminary insights into the feasibility and potential effectiveness of the intergenerational, home-based eHealth literacy intervention. This sample size allowed for a meaningful assessment of the intervention’s impact on eHealth literacy, health behaviors, and overall well-being, while also informing the design of future, larger-scale studies.

### Data Analysis

Data were initially recorded on paper and subsequently digitized using Qualtrics. Data were analyzed using IBM SPSS Statistics (version 29.0). Descriptive statistics were calculated to summarize the data, using means, standard deviations, and standard errors or frequencies and percentages as appropriate. Paired *t* tests assessed pre- to posttest changes, and Wilcoxon tests were used for nonnormal data.

To assess potential biases due to loss to follow-up, baseline characteristics of participants who completed the follow-up (n=71) were compared with those who did not (n=30) using independent sample *t* tests for continuous variables (eg, age, eHEALS) and chi-square tests for categorical variables (eg, gender, education). Missing data were addressed using multiple imputation (MI) with 50 imputations, as missing completely at random (MCAR) tests did not reject the missing at random assumption (*P*=.85 for all variables; *P*=.08 for *t* test variables) [42,43]. The MI method was chosen for its ability to reduce bias under the assumption that data are missing at random and to provide more accurate standard errors and confidence intervals [44].

The imputation model included demographic variables (eg, age, education) and several auxiliary variables, such as self-rated happiness, weekly fruit and vegetable consumption, received COVID-19 vaccine doses, and frequencies of going outside. All reported pre- and posttest values are based on MI to ensure robust findings and mitigate potential biases associated with missing data. Complete case analyses were included as sensitivity analysis. Statistical significance was set at *P*<.05.

## Results

### Recruitment and Baseline Demographics

**Figure 1.**
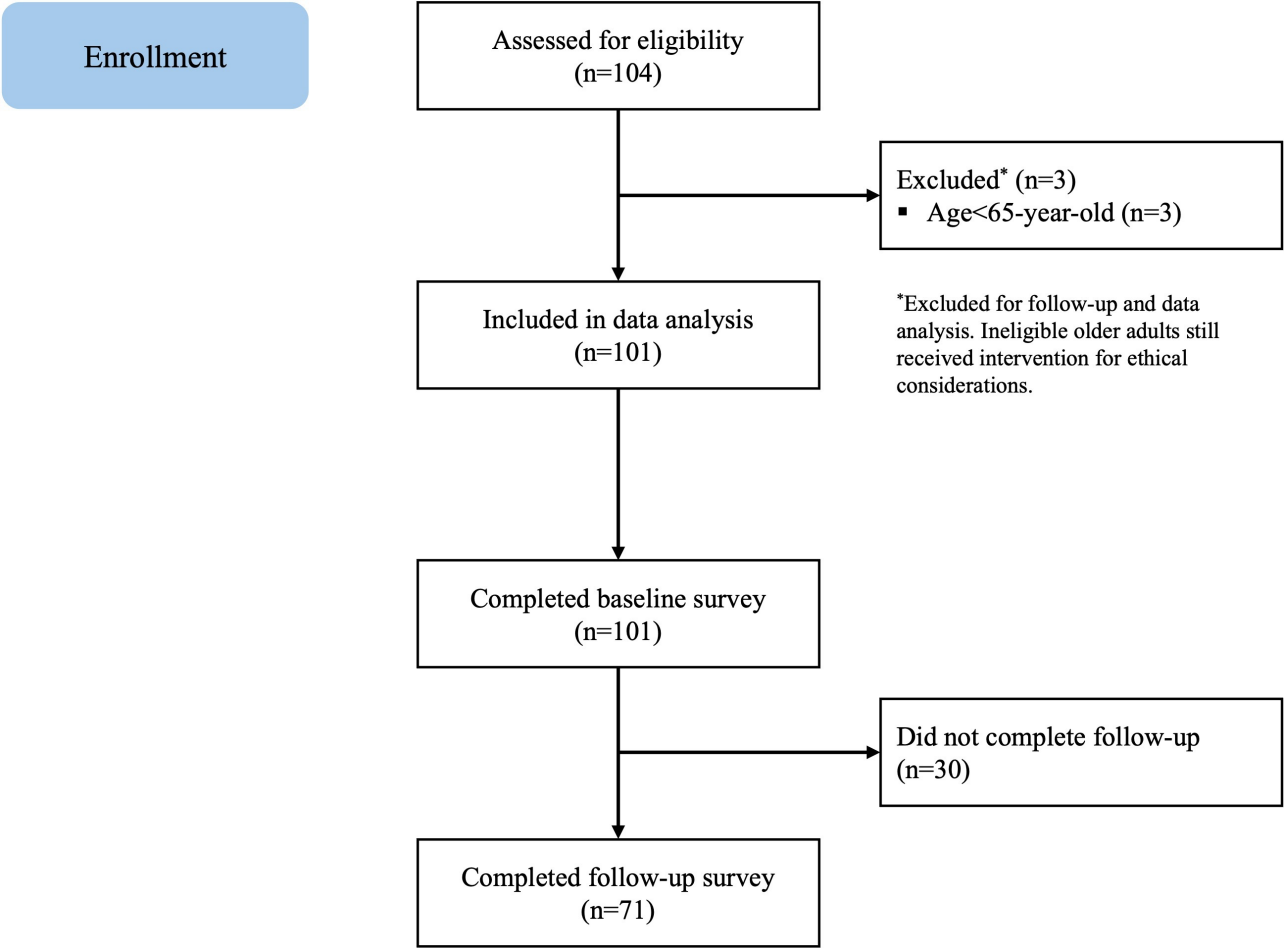
Flow diagram of participant enrollment and study procedures in the intergenerational, home-based eHealth intervention for Hong Kong’s older adult population (October-November 2022).

From the beginning of October to November 2022, 104 participants were screened as eligible; 3 (2.9%) were excluded due to being younger than 65 years, and the remaining 101 (97.1%) participants consented to the study (Figure 1). The baseline demographic characteristics of the 101 older adults are shown in Table 1. The median age was 80 (IQR 77-85) years, with the oldest participant being 93 years old. Nearly two-thirds (64/101, 63.4%) were female. Only 4.0% (4/101) reported receiving tertiary education such as college. Out of the 101 participants, 41 (40.6%) had lost their spouse, whereas 55 (54.5%) were married. Fifty-one (51.0%) participants reported living with their family. Ninety-eight (97.0%) participants reported having less than HKD $25,000 (US $3225.73) per month of household income, 42 (42.0%) reported claiming Comprehensive Social Security Assistance, and all older adults reported living in government-subsidized public housing. Out of all participants, 95 (94.1%) were retired. Physical well-being, mental well-being, and lifestyle habits of older adult participants, along with their medical history, socialization patterns, and habits, are detailed in Tables S1–S3 in (Multimedia Appendix 1). The retention rate at 2 weeks was 70.3% (71/101).

**Table 1.** Demographic and baseline characteristics of 101 older adults participating in the home-based eHealth intervention study conducted across Hong Kong’s 18 districts (October-November 2022).

Characteristic	Value
Age (years), median (IQR)	80 (77, 85)
65-74, n (%)	10 (9.9)
75-84, n (%)	64 (63.4)
≥85, n (%)	27 (26.7)
Gender, n (%)	
Male	37 (36.6)
Female	64 (63.4)
Education level (n=100; missing=1), n (%)	
Primary or below	60 (60)
Secondary	36 (36)
Tertiary	4 (4)
Marital status, n (%)	
Single	1 (1.0)
Married	55 (54.5)
Divorced	4 (4.0)
Widowed	41 (40.6)
Household income (HKD $)^a^, n (%)	
No income	62 (61.4)
<25,000	36 (35.6)
>25,000	3 (3.0)
Claiming Comprehensive Social Security Assistance (n=100; missing=1), n (%)	
No	58 (58)
Yes	42 (42)
Employment status, n (%)	
Part-time	1 (1.0)
Retired	95 (94.1)
Housewife or homemaker	5 (4.9)
Housing type	
Public Housing	101 (100)
Currently living with family (n=100; missing=1), n (%)	
Yes	49 (49)
No or no family	51 (51)
eHealth Literacy Scale (eHEALS) score (n=100; missing=1), mean (SD)	21.36 (7.85)
Owned smartphone with internet access (n=100; missing=1), n (%)	
Yes	89 (89)
No	11 (11)
Smartphone screen time per day (n=88; missing=1), mean (SD)	1.90 (1.38)
<1 hour, n (%)	55 (63)
1‐2 hours, n (%)	11 (13)
2‐3 hours, n (%)	7 (8)
3‐4 hours, n (%)	6 (7)
>4 hours, n (%)	9 (10)
Reason for using smartphone^b^ (n=88; missing=1), n (%)	
Staying in touch with family and friends	73 (83)
Watching videos	32 (36)
Browsing news	15 (17)
Listening to music	14 (16)
Gaming	10 (11)
Reading	4 (5)
Online shopping	1 (1)
Acquired or received health care–related information from online sources (n=86; missing=3), n (%)	
Yes	25 (29)
No	61 (71)
eHealth mobile apps used^b^ (n=87; missing=2), n (%)	
LeaveHomeSafe (安心出行)	84 (97)
eHealth (醫健通)	26 (30)
HA Go	9 (10)
TouchMed (e藥通)	3 (3)
None of the above	2 (2)

a HKD $1=US $0.13.

b Multiple-response questions (participant may select more than 1 choice).

### Efficacy of the Intervention

The changes in measures between baseline and the 2-week follow-up among older adults are shown in Table 2. Results from Wilcoxon rank tests (Table S4 in Multimedia Appendix 1) were consistent with the parametric *t* tests.

In terms of digital literacy and daily smartphone use, the mean eHEALS score was 21.41 (SE 0.79) at baseline and 23.80 (SE 1.41) at follow-up. This increase of 2.39 points was not statistically significant (*P*=.11). Daily smartphone usage increased by 0.45 hours, from 2.09 (SE 0.16) to 2.54 (SE 0.22) hours, which was also not significant (*P*=.07).

Among mental health measures, there was a nonsignificant decrease of 1.87 in adjusted WHO-5 scores, from 73.15 (SE 2.17) at baseline to 71.28 (SE 3.72) at follow-up (*P*=.61). The 3-item UCLA Loneliness Scale scores showed a nonsignificant increase of 0.21, from 3.92 (SE 0.13) to 4.13 (SE 0.22; *P*=.39).

For physical well-being and activity measures, self-rated physical well-being had a nonsignificant change from 3.51 (SE 0.09) at baseline to 3.67 (SE 0.19) at follow-up (*P*=.42). Changes in exercise variables were not significant either; strenuous exercise increased from 47.41 (SE 12.66) to 65.88 (SE 17.91; *P*=.36) minutes per week, moderate exercise decreased from 171.12 (SE 26.77) to 153.94 (SE 29.02; *P*=.61) minutes per week, and light exercise (walking) increased from 525.92 (SE 58.92) to 773.00 (SE 153.80; *P*=.13) minutes per week. Sitting, measured in minutes per day, had a nonsignificant increase from 367.32 (SE 26.14) to 398.09 (SE 52.93; *P*=.57).

All complete case results were similar to those obtained using MI, except for self-rated physical well-being. Complete case analyses showed a significant increase in physical well-being ratings, from 3.61 (SE 0.11) to 3.82 (SE 0.09; *P*=.04).

At the 2-week follow-up, 70% (50/71) of older adults reported exercising more frequently, 61% (43/71) reported watching health-related videos more often, and 55% (39/71) washed their hands more regularly (Table S5 in Multimedia Appendix 1).

**Table 2.** Primary and secondary outcomes of the intergenerational, home-based eHealth intervention for Hong Kong’s older adult population (October-November 2022).

Variables	Participants, n	Baseline, mean (SE)	2-week follow-up, mean (SE)	*P* value	Cohen *d*
Digital health literacy and smartphone use					
eHEALS^a^					
MI^b^	101	21.41 (0.79)	23.80 (1.41)	.11	—^d^
CC^c^	70	21.53 (0.93)	23.40 (1.02)	.10	0.20
Screen time^e^					
MI	101	2.09 (0.16)	2.54 (0.22)	.07	—
CC	64	2.06 (0.18)	2.13 (0.15)	.71	0.05
Additional measures					
WHO-5^f^					
MI	101	73.15 (2.17)	71.28 (3.72)	.61	—
CC	71	73.80 (2.68)	73.80 (2.51)	≥.99	0.00
UCLA-3^g^					
MI	101	3.92 (0.13)	4.13 (0.22)	.39	—
CC	70	3.99 (0.16)	3.90 (0.19)	.69	–0.05
Physical well-being^h^					
MI	101	3.51 (0.09)	3.67 (0.19)	.42	—
CC	71	3.61 (0.11)	3.82 (0.09)	.04	0.25
Strenuous exercise^i^					
MI	101	47.41 (12.66)	65.88 (17.91)	.36	—
CC	68	45.81 (12.76)	38.81 (11.72)	.61	–0.06
Moderate exercise^i^					
MI	101	171.12 (26.77)	153.94 (29.02)	.61	—
CC	70	168.86 (31.41)	123.70 (22.07)	.08	–0.21
Walking^i^					
MI	101	525.92 (58.92)	773.00 (153.80)	.13	—
CC	68	496.01 (64.36)	548.38 (88.38)	.63	0.06
Sitting^j^					
MI	101	367.32 (26.14)	398.09 (52.93)	.57	—
CC	68	381.18 (32.23)	358.24 (30.97)	.46	–0.09

a eHEALS: eHealth Literacy Scale.

b MI: multiple imputation.

c CC: complete case.

d Not applicable.

e Time (categorical hours) spent using smartphone per day.

f WHO-5: World Health Organization–Five Well-Being Index.

g UCLA-3: 3-item University of California, Los Angeles Loneliness Scale.

h Self-reported single score, ranging from 1 to 5 (1= very poor, 5=very good).

i Minutes per week.

j Minutes per day.

### Feasibility and Acceptability of the Intervention

At baseline, 57 university student interventionists delivered material (n=101, 100%) on eHealth literacy, NCD prevention, psychosocial health, COVID-19 prevention, and educational leaflets. They demonstrated QR code scanning, encouraged independent practice, assisted with installing health apps and registering for services, and created one-on-one WhatsApp group chats with older adults after the intervention (n=101, 100%).

At the 2-week follow-up, participants self-reported their mean satisfaction with the intervention and the 14-day WhatsApp messaging period as 4.32 (SD 0.53) and 3.87 (SD 0.72), respectively, out of 5.

### Other Differences

No significant differences were found between participants who completed the follow-up (n=71) and those who did not (n=30) in age, gender, income, or living arrangement. A significant difference was observed in education level, but it should be noted that this observation was made among a lower number of participants (n<5) at times (Table S7 in Multimedia Appendix 1).

## Discussion

### Principal Findings

This pilot study indicates that an intergenerational, home-based eHealth intervention package is a feasible and acceptable method of improving older adults’ engagement with digital health resources while promoting healthy behaviors. These findings address our objective to examine the feasibility and effectiveness of such an intervention in Hong Kong’s older adult population.

The intervention showed promising, though modest, improvements in eHealth literacy and smartphone usage among older adults. The mean eHEALS scores increased by 2.39 (21.41, SE 0.79 to 23.80, SE 1.41) points, and daily smartphone use rose by 0.45 hours (2.09 to 2.54 hours), though these changes were not statistically significant. Despite the lack of statistical significance, the positive trends suggest potential benefits that warrant further investigation with larger sample sizes and longer follow-up periods. The high satisfaction rating (4.32 out of 5) indicates strong acceptability among participants. Furthermore, most participants reported improved health behaviors, including increased physical activity (50/71, 70%), more frequent viewing of health videos (43/71, 61%), and improved handwashing practices (39/71, 55%), indicating the intervention positively impacted their daily routines and health.

Comparisons with existing literature reveal interesting patterns. Our findings align with previous studies [21,22], but the comparisons suggest that longer durations may be required to achieve more substantial improvements in digital literacy. For example, Xie’s study [21] involved 2 weekly classes over a total of 4 weeks and showed significant improvements in participants’ computer knowledge (*P*<.01). Additionally, our sample was noticeably older (mean age: 80.42, SD 5.72 years) with lower baseline eHEALS scores (mean 21.41, SE 0.79) compared to that of Chang et al [22], whose participants (mean age: 74.64 years) started with higher baseline eHEALS scores (mean 28.31) and achieved statistically significant improvements 2 months postintervention (*P*<.001). These comparisons suggest that the older age and limited digital experience of our participants may have constrained the impact of the intervention within the 2-week timeframe. Previous literature [45,46] suggests that longer follow-up time periods may be required to observe noticeable changes as older adults require time to learn and put knowledge to use.

The home-based design of the intervention was effective in reaching underprivileged older adults, a population often overlooked by traditional classroom-based programs. Only 4.0% (4/101) of participants had tertiary education, and just 3.0% (3/101) had a household income above the local median (HKD $25,000 per month). Many participants had limited prior exposure to eHealth technologies; fewer than 30% had ever searched for health information online, and most had not used mobile health apps, except for the government-mandated COVID-tracking app, LeaveHomeSafe. Conducting the intervention in participants’ homes allowed them to learn comfortably at their own pace, free from the fear of judgment by peers, an essential factor given their low baseline eHEALS scores (mean 21.41, SE 0.79). The home-based setting also helped minimize stress and boost confidence, enhanced by positive language and a personalized pace set by student interventionists. This approach removed several key barriers commonly identified in older adults’ learning of digital technologies [20].

We also recruited 57 nursing students (Table S6 in Multimedia Appendix 1) with varying levels of study and background knowledge, all of whom received specialized training covering primary health care, aging populations in Hong Kong, health assessments, intervention delivery, and communication skills with older adults. This experience not only provided student interventionists with an extracurricular opportunity to develop practical skills but also enhanced their ability to communicate with older adults, an important component of geriatric care. Programs like the Generations Connect Project demonstrate the potential of intergenerational interventions to support older adults while enriching health care students’ education and professional development [14].

This study had 3 limitations. First, the short follow-up period may have limited our ability to observe significant changes in health-related indicators and eHealth literacy. Future studies with longer follow-up periods are needed to better assess the sustained effects of interventions. Second, the follow-up surveys were conducted by telephone due to privacy concerns, differing from the home-based baseline assessments, which may have affected consistency. Third, the low educational attainment of participants (60/101, 60.0% having received only primary school education or below) may have hindered their engagement with the intervention content, reducing its effectiveness. These limitations highlight the need for materials tailored to the educational level of older adults and for more standardized intervention protocols in future research.

### Conclusions

This pilot study showed that an intergenerational, home-based eHealth intervention may be an effective approach to improving older adults’ engagement with digital health care resources while simultaneously supporting their physical and psychological well-being. The findings suggest that such interventions can help bridge the digital divide for older adult populations and promote healthier behaviors.

Future research should consider extending the intervention duration, increasing the frequency of home visits, and developing more tailored educational materials. Additionally, collaborations with local health care agencies could help address financial barriers by providing affordable mobile devices and internet access. The broader implications of this research extend to health care policy and practice, highlighting the potential of community-based, intergenerational approaches to address the growing health care needs of aging populations while preparing health care students for geriatric care through practical, community-oriented training experiences.

## Supplementary material

10.2196/69611 Multimedia Appendix 1 Supplementary tables on additional participant characteristics and demographic information.

10.2196/69611 Checklist 1 CONSORT 2010 checklist.

## Abbreviations

CONSORT: Consolidated Standards of Reporting Trials
eHEALS: eHealth Literacy Scale
GDP: gross domestic product
MCAR: missing completely at random
MI: multiple imputation
NCD: noncommunicable disease
UCLA: University of California, Los Angeles

## References

[1] (2023). Hong Kong 2021 Population Census - thematic report: older persons. https://www.censtatd.gov.hk/en/EIndexbySubject.html?scode=600&pcode=B1120118.

[2] (2023). Hong Kong population projections 2022-2046. https://www.censtatd.gov.hk/en/data/stat_report/product/B1120015/att/B1120015092023XXXXB01.pdf.

[3] Choi M, Sempungu JK, Lee EH, Lee YH (2024). Living longer but in poor health: healthcare system responses to ageing populations in industrialised countries based on the findings from the Global Burden of Disease Study 2019. BMC Public Health.

[4] (2021). Global expenditure on health: public spending on the rise. https://www.who.int/publications/i/item/9789240041219.

[5] (2024). Hong Kong country commercial guide. International Trade Adminstration.

[6] Yip WCM, Hsiao WC, Chen W, Hu S, Ma J, Maynard A (2012). Early appraisal of China’s huge and complex health-care reforms. Lancet.

[7] Du X, Patel A, Anderson CS, Dong J, Ma C (2019). Epidemiology of cardiovascular disease in China and opportunities for improvement: JACC International. J Am Coll Cardiol.

[8] (2023). Coping with chronic illness. Elderly Health Service Department of Health.

[9] Kim J, Kim Y, Ha J (2021). Changes in daily life during the COVID-19 pandemic among South Korean older adults with chronic diseases: a qualitative study. Int J Environ Res Public Health.

[10] Petrova NN, Khvostikova DA (2021). Prevalence, structure, and risk factors for mental disorders in older adults. Adv Gerontol.

[11] Sinha P, Hussain T, Boora NK (2021). Prevalence of common mental disorders in older adults: results from the National Mental Health Survey of India. Asian J Psychiatr.

[12] Zenebe Y, Akele B, W/Selassie M, Necho M (2021). Prevalence and determinants of depression among old age: a systematic review and meta-analysis. Ann Gen Psychiatry.

[13] Vernooij-Dassen M, Verhey F, Lapid M (2020). The risks of social distancing for older adults: a call to balance. Int Psychogeriatr.

[14] Choi EY, Farina MP, Wu Q, Ailshire J (2022). COVID-19 social distancing measures and loneliness among older adults. J Gerontol B Psychol Sci Soc Sci.

[15] Ernst M, Niederer D, Werner AM (2022). Loneliness before and during the COVID-19 pandemic: a systematic review with meta-analysis. Am Psychol.

[16] Norman CD, Skinner HA (2006). eHealth literacy: essential skills for consumer health in a networked world. J Med Internet Res.

[17] Berkowsky RW (2021). Exploring predictors of eHealth literacy among older adults: findings from the 2020 CALSPEAKS survey. Gerontol Geriatr Med.

[18] Frishammar J, Essén A, Bergström F, Ekman T (2023). Digital health platforms for the elderly? Key adoption and usage barriers and ways to address them. Technol Forecast Soc Change.

[19] Hunsaker A, Hargittai E (2018). A review of internet use among older adults. New Media & Society.

[20] Pourrazavi S, Kouzekanani K, Bazargan-Hejazi S (2020). Theory-based e-health literacy interventions in older adults: a systematic review. Arch Public Health.

[21] Xie B (2012). Improving older adults’ e-health literacy through computer training using NIH online resources. Libr Inf Sci Res.

[22] Chang SJ, Yang E, Lee KE, Ryu H (2021). Internet health information education for older adults: a pilot study. Geriatr Nurs.

[23] Chiu CJ, Hu YH, Lin DC, Chang FY, Chang CS, Lai CF (2016). The attitudes, impact, and learning needs of older adults using apps on touchscreen mobile devices: results from a pilot study. Comput Human Behav.

[24] Opdenacker J, Boen F, Coorevits N, Delecluse C (2008). Effectiveness of a lifestyle intervention and a structured exercise intervention in older adults. Prev Med.

[25] Turner J, Greenawalt K, Goodwin S, Rathie E, Orsega-Smith E (2017). The development and implementation of the Art of Happiness intervention for community-dwelling older adults. Educ Gerontol.

[26] Arthanat S, Vroman KG, Lysack C, Grizzetti J (2019). Multi-stakeholder perspectives on information communication technology training for older adults: implications for teaching and learning. Disabil Rehabil Assist Technol.

[27] Geerts N, Schirmer W, Vercruyssen A, Glorieux I (2023). Bridging the ‘instruction gap’: how ICT instructors help older adults with the acquisition of digital skills. Int J Lifelong Educ.

[28] LoBuono DL, Leedahl SN, Maiocco E (2020). Teaching technology to older adults: modalities used by student mentors and reasons for continued program participation. J Gerontol Nurs.

[29] Pihlainen K, Ehlers A, Rohner R (2023). Older adults’ reasons to participate in digital skills learning: an interdisciplinary, multiple case study from Austria, Finland, and Germany. Studies in the Education of Adults.

[30] Chang SJ, Choi S, Kim SA, Song M (2014). Intervention strategies based on Information-Motivation-Behavioral Skills model for health behavior change: a systematic review. Asian Nurs Res (Korean Soc Nurs Sci).

[31] Fisher WA, Fisher JD, Harman J (2003). Social Psychological Foundations of Health and Illness.

[32] Phang JK, Kwan YH, Yoon S (2023). Digital intergenerational program to reduce loneliness and social isolation among older adults: realist review. JMIR Aging.

[33] Bae S, Kim D (2024). Improving home-like environments in long-term care units: an exploratory mixed-method study. Sci Rep.

[34] (2024). Generations Connect. HKU Bulletin.

[35] Eldridge SM, Chan CL, Campbell MJ (2016). CONSORT 2010 statement: extension to randomised pilot and feasibility trials. BMJ.

[36] Kim S, Chow BC, Park S, Liu H (2023). The usage of digital health technology among older adults in Hong Kong and the role of technology readiness and ehealth literacy: path analysis. J Med Internet Res.

[37] Xie L, Mo PKH (2023). Comparison of eHealth Literacy Scale (eHEALS) and Digital Health Literacy Instrument (DHLI) In assessing electronic health literacy in Chinese older adults: a mixed-methods approach. Int J Environ Res Public Health.

[38] Jiang X, Wang L, Leng Y (2024). The level of electronic health literacy among older adults: a systematic review and meta-analysis. Arch Public Health.

[39] Topp CW, Østergaard SD, Søndergaard S, Bech P (2015). The WHO-5 Well-Being Index: a systematic review of the literature. Psychother Psychosom.

[40] Russell DW (1996). UCLA Loneliness Scale (version 3): reliability, validity, and factor structure. J Pers Assess.

[41] Craig CL, Marshall AL, Sjöström M (2003). International physical activity questionnaire: 12-country reliability and validity. Med Sci Sports Exerc.

[42] Kang H (2013). The prevention and handling of the missing data. Korean J Anesthesiol.

[43] Little RJA (1988). A test of missing completely at random for multivariate data with missing values. J Am Stat Assoc.

[44] Woods AD, Gerasimova D, Van Dusen B (2024). Best practices for addressing missing data through multiple imputation. Infant Child Dev.

[45] Göransson C, Wengström Y, Hälleberg-Nyman M, Langius-Eklöf A, Ziegert K, Blomberg K (2020). An app for supporting older people receiving home care - usage, aspects of health and health literacy: a quasi-experimental study. BMC Med Inform Decis Mak.

[46] Nahm ES, Zhu S, Bellantoni M (2019). The effects of a theory-based patient portal e-learning program for older adults with chronic illnesses. Telemed J E Health.

