# Supplementary material for: Boosting Digital Health Engagement Among Older Adults in Hong Kong: Pilot Pre-Post Study of the Generations Connect Project
Source: JMIR Form Res. 2025 May 8;9:e69611. doi: 10.2196/69611 (PMC12080964; doi:10.2196/69611)
Supplement: Multimedia Appendix 1 [file formative-v9-e69611-s001.docx]

**Multimedia Appendix of supplementary files: Appendix Tables**

Table S1. Physical Wellbeing and Medical History of Older Adults Participating in the Intergenerational Home-based eHealth Intervention in Hong Kong (October–November 2022)

| Characteristic | N (%) | Missing data, N |
| --- | --- | --- |
| **Self-rated Physical Wellbeing** |  | **0** |
| *Mean (SD)* | *3.51 (.9%)* |  |
| Very Poor (1) | 0 |  |
| Poor (2) | 15 (14.9%) |  |
| Fair (3) | 33 (32.7%) |  |
| Good (4) | 39 (38.6%) |  |
| Very Good (5) | 14 (13.9%) |  |
| Did *NOT* Have Any Chronic Diseases | 5 (5.0%) |  |
| **COVID-19 Contraction** |  | **1** |
| Yes | 30 (30.0%) |  |
| No or Do not Know | 70 (70.0%) |  |
| **Received COVID-19 Vaccine** |  | **1** |
| Yes | 94 (94%) |  |
| No | 6 (6%) |  |
| **If Vaccinated: Dosage Received (n= 94)** |  | **0** |
| 1 Dose | 1 (1%) |  |
| 2 Doses | 4 (4%) |  |
| 3 Doses | 67 (71%) |  |
| 4 Doses | 21 (22%) |  |
| *Could Not Remember* | *1 (1%)* |  |
| **Medical Services Accessed^a^** |  | **2** |
| Public Hospital | 90 (90.9%) |  |
| District Health Center | 21 (21.2%) |  |
| Department of Health | 11 (11.1%) |  |
| **Location for Medical Follow-ups^a^** |  | **7** |
| Hospital Authority | 89 (94.7%) |  |
| Private Doctor | 18 (19.1%) |  |
| Others | 3 (3.2%) |  |

**^a^**Multiple Response Questions (Subject may select more than one choice)

Table S2. Mental Wellbeing and Socialization Patterns of Older Adults in the Intergenerational Home-based eHealth Intervention Study Conducted Across Hong Kong's 18 Districts (October–November 2022)

| Characteristic | N (%) | Missing data, N |
| --- | --- | --- |
| **WHO-5 Mental Wellbeing Index** |  | **0** |
| *Mean (SD)* | *73.15 (21.82)* |  |
| **UCLA-3 Loneliness Scale** |  | **1** |
| *Mean (SD)* | *3.92 (1.34)* |  |
| **Communication Methods^a^** |  | **0** |
| Telephone | 76 (75.2%) |  |
| Face-to-face | 66 (65.3%) |  |
| WhatsApp (Instant Messaging Mobile App) | 36 (35.6%) |  |
| **Self-rated Family Happiness^b^** |  | **0** |
| *Mean (SD)* | 7.65 (2.09) |  |
| **Having face-to-face communication almost every single day with^a^** |  | **0** |
| Family Members | 59 (58.4%) |  |
| Friends | 39 (38.6%) |  |
| Neighbors | 29 (28.7%) |  |
| *No Face-to-face available* | *11 (10.9%)* |  |
| **Frequency of Going Outside, Days per Week** |  | **1** |
| *Mean (SD)* | *5.13 (2.12)* |  |
| **Activities When Going Outside^a^** |  | **0** |
| Buying Necessities | 78 (77.2%) |  |
| Dining | 46 (45.5%) |  |
| Exercising | 45 (44.6%) |  |
| Visiting Doctor | 39 (38.6%) |  |
| Socializing with Families and Friends | 36 (35.6%) |  |

**^a^**Multiple Response Questions (Subject may select more than one choice)

**^b^**Self-reported family happiness (range: 0 to 10). A higher score means a happier family.

Table S3. Lifestyle and Habits of Older Adults Participating in the Intergenerational Home-based eHealth Intervention Study in Hong Kong (October–November 2022)

| Characteristic | Mean (SD) | Missing data, N |
| --- | --- | --- |
| **Current Tobacco Users**, N (%) | 4 (4.0%) | **0** |
| **Consumed Alcohol at Least Once per Month**, N (%) | 11 (10.9%) | **0** |
| **Frequently Eat Fatty Meat**, N (%) | 6 (5.9%) | **0** |
| **Frequently Take Extra Seasoning**, N (%) | 8 (8.0%) | **0** |
| **Food Consumed, Portions per Week** |  |  |
| Sugary Drinks | 1.65 (2.68) | **0** |
| Vegetables | 9.70 (5.61) | **36** |
| Fruits | 7.33 (4.51) | **1** |
| **Exercises, Minutes per Week^a^** |  |  |
| Strenuous Exercise | 42.76 (123.48) | **5** |
| Moderate Exercise | 161.12 (256.03) | **3** |
| Walking (Light Exercise) | 515.91 (578.12) | **3** |
| **Sitting, Minutes per Day^a^** | 369.12 (259.49) | **4** |

**^a^** Measured by International Physical Activity Questionnaire - Short Form

Table S4. Baseline (Pre) and 2-week Follow-up (Post) Outcomes of the Intergenerational Home-based eHealth Intervention for Hong Kong's Elderly Population (October–November 2022): Wilcoxon Rank Test Results (Complete-Case Analysis)

| Variables | Pre to Post Ranks |  |  |  |  |  |
| --- | --- | --- | --- | --- | --- | --- |
| (Total N) |  | *N* | *Mean Rank* | *Sum of Ranks* | *Z* | *P-value*^b^ |
| WHO-5 | Negative | 23 | 24.72 | 568.50 | -.20 | .84 |
| (71) | Positive | 25 | 24.30 | 607.50 |  |  |
|  | Ties | 23 |  |  |  |  |
| UCLA3 | Negative | 21 | 16.38 | 344.00 | -.81 | .42 |
| (70) | Positive | 13 | 19.31 | 251.00 |  |  |
|  | Ties | 36 |  |  |  |  |
| Screentime | Negative | 14 | 23.36 | 327.00 | -.38 | .70 |
| (64) | Positive | 23 | 16.35 | 376.00 |  |  |
|  | Ties | 27 |  |  |  |  |
| eHEALS | Negative | 22 | 28.00 | 616.00 | -1.86 | .06 |
| (70) | Positive | 36 | 30.42 | 1095.00 |  |  |
|  | Ties | 12 |  |  |  |  |
| Physical Wellbeing**^a^** | Negative | 10 | 13.95 | 139.50 | -2.04 | .04 |
| (71) | Positive | 20 | 16.28 | 325.50 |  |  |
|  | Ties | 41 |  |  |  |  |
| Strenuous Exercise | Negative | 11 | 14.95 | 164.50 | -.05 | .96 |
| (68) | Positive | 14 | 11.46 | 160.50 |  |  |
|  | Ties | 43 |  |  |  |  |
| Moderate Exercise | Negative | 23 | 23.96 | 551.00 | -1.25 | .21 |
| (70) | Positive | 19 | 18.53 | 352.00 |  |  |
|  | Ties | 28 |  |  |  |  |
| Walking | Negative | 26 | 27.37 | 711.50 | -.205 | .84 |
| (68) | Positive | 26 | 25.63 | 666.50 |  |  |
|  | Ties | 16 |  |  |  |  |
| Sitting | Negative | 27 | 31.07 | 839.00 | -.84 | .40 |
| (68) | Positive | 27 | 23.93 | 646.00 |  |  |
|  | Ties | 14 |  |  |  |  |

Note*.* ^a^*P*< .05.

^b^ Asymptotic Significance (2-tailed).

Table S5. Perceived Frequencies of Performing Health-Promoting Behaviors Among Participants in the Intergenerational Home-based eHealth Intervention Study in Hong Kong (N=71), Compared with Pre-Intervention Frequencies (October–November 2022)

| Items | Much Less | Less | No Change | More | Much More |
| --- | --- | --- | --- | --- | --- |
|  |  |  | *N (%)* |  |  |
| Physical Exercise^a^ | 0 | 0 (1.3%) | 21 (29.6%) | 40 (56.3%) | 10 (14.1%) |
| Watching Videos^b^ | 1 (1.4%) | 3 (4.2%) | 24 (33.8%) | 31 (43.7%) | 12 (16.9%) |
| Washing Hands^c^ | 3 (4.2%) | 2 (2.8%) | 27 (38.0%) | 26 (36.6%) | 13 (18.3%) |

^a^ Physical Exercise: Changes in frequency of performing physical exercises, specifically exercises tailored to older adults’ physical abilities, taught by and practiced with student ambassadors during home visits.

^b^ Watching Videos: Change in frequency of watching health-related videos from online sources using their smartphones.

^c^ Washing Hands: Change in frequency of properly washing hands by adhering to the “5-step” hand-washing protocol introduced by and practiced with student ambassadors during home visits.

Table S6. Demographics of Student Ambassadors Involved in the Intergenerational Home-based eHealth Intervention Study in Hong Kong (N=57, October–November 2022)

| **Characteristic** |  | N | % |
| --- | --- | --- | --- |
| **Gender** |  |  |  |
| Male |  | 23 | 40.4% |
| Female |  | 34 | 59.6% |
| **Age** |  |  |  |
| Below 18 |  | 2 | 3.5% |
| 18 – 21 |  | 37 | 64.9% |
| 22 – 25 |  | 16 | 28.1% |
| 26 – 28 |  | 1 | 1.8% |
| 29 or above |  | 1 | 1.8% |
| **Year of Program** |  |  |  |
| Bachelor of Nursing, Year 1 |  | 24 | 42.1% |
| Bachelor of Nursing, Year 2 |  | 7 | 12.3% |
| Bachelor of Nursing, Year 3 |  | 8 | 14.0% |
| Bachelor of Nursing, Year 4 |  | 6 | 10.5% |
| Bachelor of Nursing, Year 5 |  | 6 | 10.5% |
| Master of Science in Nursing, Year 1 |  | 6 | 10.5% |
| **Education Attained** |  |  |  |
| HKDSE (High School Diploma) |  | 39 | 68.4% |
| Associate’s degree |  | 11 | 19.3% |
| Bachelor’s degree in Other Programs |  | 7 | 12.3% |
| **Currently Living with Parents or Grandparents** |  |  |  |
| Yes |  | 53 | 93.0% |
| No |  | 4 | 7.0% |
| **Having Experience Providing Service to Older Adults** |  |  |  |
| Yes |  | 26 | 45.6% |
| No |  | 31 | 54.4% |
| **Attended Primary Healthcare Practicum** |  |  |  |
| Yes |  | 20 | 35.1% |
| No |  | 37 | 64.9% |

Table S7. Comparison of Baseline Characteristics Between Participants Who Completed Follow-up and Those Who Missed Follow-up in the Intergenerational Home-based eHealth Intervention Study in Hong Kong (October–November 2022)

| Variables | Completed follow-up  N=71 | | Missed follow-up  N=30 | *P*-value |
| --- | --- | --- | --- | --- |
| Age (Mean, SD) ^a^ | | 80.03 (6.25) | 80.80 (5.31) | .56 |
| Age as categories (N, %) ^b^ | |  |  | .76 |
| Young-old (65-74) | | 6  60.0% | 4  40.0% |  |
| Mid-old (75-84) | | 45  71.4% | 18  28.6% |  |
| Oldest-old (85+) | | 19  70.4% | 8  29.6% |  |
| Gender (N, %) ^b^ | |  |  | .75 |
| Male | | 26  68.4% | 12  31.6% |  |
| Female | | 45  71.4% | 18  28.6% |  |
| Education ^b^ | |  |  | .005 |
| Primary or below | | 36  61.0% | 23  39.0% |  |
| Secondary | | 32  88.9% | 4  11.1% |  |
| Tertiary | | 2  40.0% | 3  60.0% |  |
| Income ^b^ | |  |  | .30 |
| No income | | 41  65.1% | 22  34.9% |  |
| <$25,000 HKD | | 28  80.0% | 7  20.0% |  |
| >= $25,000 HKD | | 2  66.7% | 1  33.3% |  |
| Living with family? ^b^ | |  |  | .71 |
| Yes, living with family | | 33  67.3% | 16  32.7% |  |
| No, not living with family | | 36  72.0% | 14  28.0% |  |
| No family | | 1  100.0% | 0  0% |  |

^a^ Independent samples t-test

^b^ Chi-square test of independence
